# Fast Estimation of Strains for Cross-Beams Six-Axis Force/Torque Sensors by Mechanical Modeling

**DOI:** 10.3390/s130506669

**Published:** 2013-05-17

**Authors:** Junqing Ma, Aiguo Song

**Affiliations:** Jiangsu Key Lab of Remote Measurement and Control, School of Instrument Science and Engineering, Southeast University, Nanjing 210096, China; E-Mail: mjqseu@gmail.com

**Keywords:** mechanical model, cross beams, six-axis force/torque sensors, FEA, strains

## Abstract

Strain distributions are crucial criteria of cross-beams six-axis force/torque sensors. The conventional method for calculating the criteria is to utilize Finite Element Analysis (FEA) to get numerical solutions. This paper aims to obtain analytical solutions of strains under the effect of external force/torque in each dimension. Genetic mechanical models for cross-beams six-axis force/torque sensors are proposed, in which deformable cross elastic beams and compliant beams are modeled as quasi-static Timoshenko beam. A detailed description of model assumptions, model idealizations, application scope and model establishment is presented. The results are validated by both numerical FEA simulations and calibration experiments, and test results are found to be compatible with each other for a wide range of geometric properties. The proposed analytical solutions are demonstrated to be an accurate estimation algorithm with higher efficiency.

## 1. Introduction

Cross-beams six-axis force/torque sensors are typical six-axis force/torque sensors used in various branches of engineering, especially in robotic manipulators, for their compactness, simplicity and low interference errors. A six-axis force/torque sensor is usually mounted between the distal end of a robot arm and an end-effector to measure the interaction Cartesian forces and torques (*F_x_*, *F_y_*, *F_z_* and *M_x_*, *M_y_*, *M_z_*) between the robot arm and the environment [1–3]. The cross-beams elastic body (elastic body) is the key component of a six-axis force/torque sensor. The brief measuring principle of cross-beams six-axis force sensors can be described as follows. The elastic body will be deformed under external forces/toques. Strain gauges are pasted firmly on surfaces of cross elastic beams in order to detect deformations and convert variations of strains to variations of electric resistances. Finally, changes of electric resistances are converted into six-dimensional output voltages by Wheatstone bridges [4–7].

**Figure 1.**
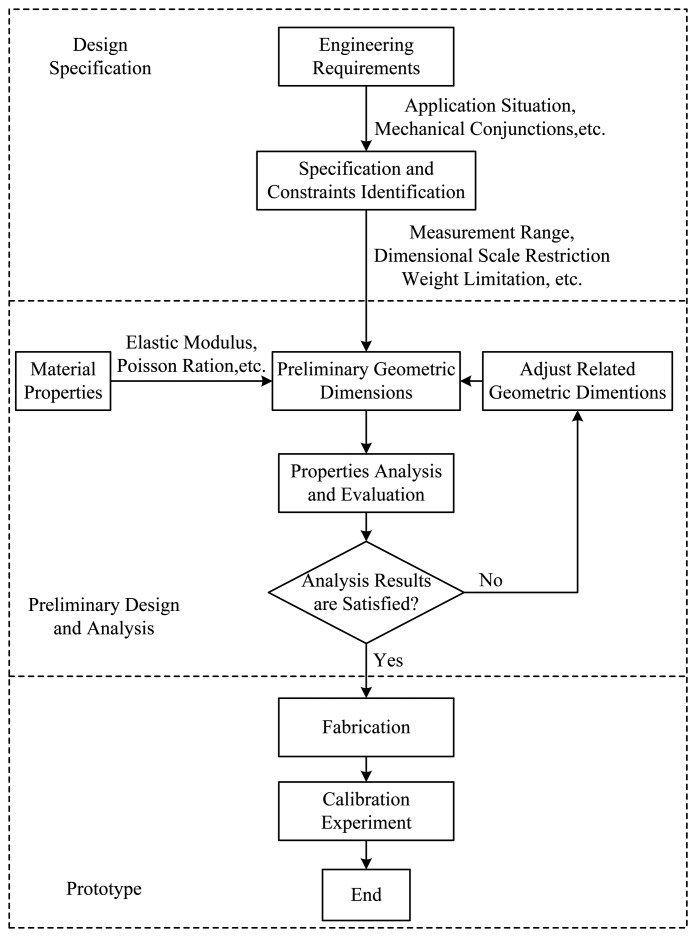
Design process of six-axis force/toque sensors.

In the design process, geometric dimensions of an elastic body need to be adjusted taking into consideration design specifications and constraints obtained from engineer requirements, such as the measurement range, weight and dimensional restrictions. Once a preliminary design of an elastic body is proposed, many structure properties need to be analyzed, such as strain distributions of the elastic body under external force/torque in each dimension, and locations where strain gauges should be attached [7,8]. If an acceptable structure is conceived, a prototype is fabricated, and calibration experiments are used to test performances of the elastic body. Figure 1 depicts a flow chart of the whole design process. The conventional method to evaluate the above properties of an elastic body is to use FEA by commercial FEA software, like ANSYS or ABAQUS, to get numerical solutions. With this method, Song *et al.* in [9] developed a four-axis cross-beams force sensor for HapticHCI with a measurement range of 20 N and 4.5 Nmm. Chen *et al.* in [10] designed a three-axis cross-beams force sensor with a measurement range of 200 N. Ma *et al.* in [11] designed a two-axis cross-beams force sensor for massage robots with a measurement range of 100 N. However, as the FEA is time consuming, it always takes quite a while to calculate strain distributions of a cross-beams elastic body with a certain set of geometric parameters under effects of forces/torques in all directions. Moreover, if the geometric parameters of an elastic body need to be adjusted several times before final judgment can be made as to which structural form is most appropriate, then the whole calculation takes up much longer time. Consequently, there is a need to design a faster calculation method to improve the design efficiency of cross-beams elastic bodies.

In this paper, we propose closed-form expressions to fast estimate strain distributions of cross-beams six-axis force sensors based on the establishment of mechanical models. In the proposed mechanical models, all deformable beams of elastic bodies are idealized as deep beams or short beams, which are supported and connected together in various methods. The simplified mechanical models are analyzed by Timoshenko beam theory. The Timoshenko beam theory takes into account shear deformation and rotational inertial effects, making it more suitable and accurate to describe behaviors of deep beams and short beams than the Euler–Bernoulli beam theory (also known as classic beam theory). Analytical predictions are subsequently validated by FEA simulations and calibration experiments. Our method is proven to provide a reliable means of calculating strains with higher efficiency.

## 2. Mechanical Structure and Distribution of Strain Gauges

**Figure 2.**
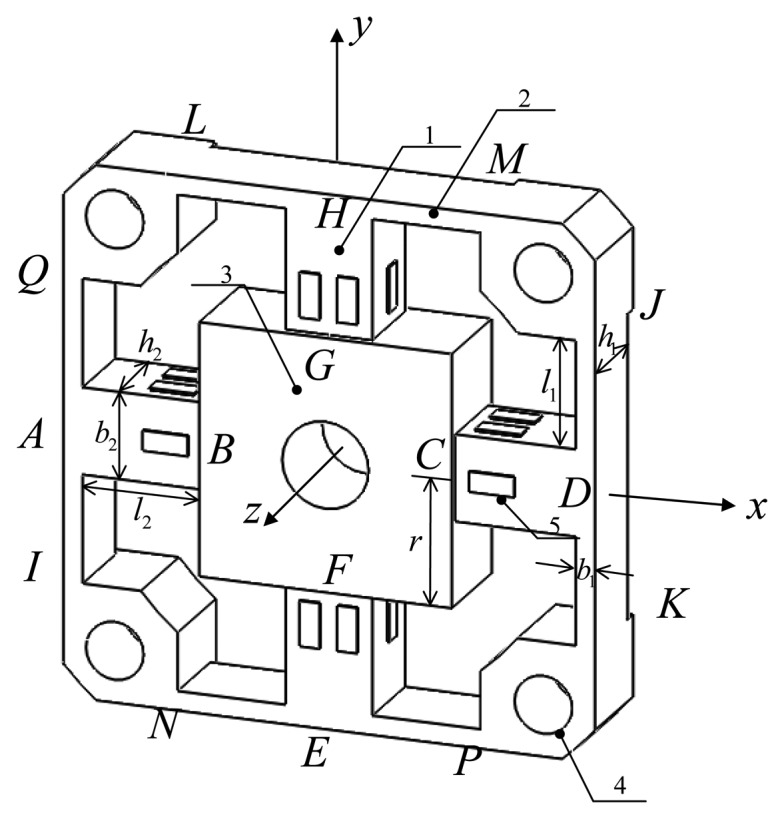
Geometric characteristics of the elastic body. (**1**) Cross elastic beams; (**2**) Compliant beams; (**3**) Square convex; (**4**) Location holes; (**5**) Strain gauges.

Figure 2 depicts mechanical notations and dimensions of a typical cross-beams elastic body in global coordinate. The elastic body contains four cross elastic beams (*i.e.*, *AB*, *CD*, *EF*, *GH*), eight compliant beams (*i.e.*, *AQ*, *AI*, *HL*, *HM*, *DJ*, *DK*, *EP*, *EN*), a square convex and four location holes. Twenty-four uniaxial strain gauges are pasted on surfaces of each cross elastic beam to measure related strain values. Each four strain gauges are connected to form a full Wheatstone bridge according to six distinct deformation conditions of the elastic body under effects of each one-dimensional force/torque, *i.e.*, *F_x_*, *F_y_*, *F_z_* and *M_x_*, *M_y_*, *M_z_*. Parameters *l*_1_, *b*_1_, *h*_1_ denote the length, width and height of compliant beams, and parameters *l*_2_, *b*_2_, *h*_2_ denote the length, width and height of cross elastic beams, respectively. In most cases, *b*_2_ is equal to *h*_2_. The parameter *r* represents half of the width or length of the square convex.

In an actual force perception task, external forces/torques are applied on the square convex and transmitted to cross elastic beams. The cross elastic beams will subsequently be deformed. Compliant beams, which are flexible to external forces/torques too, can be regarded as elastic supports or floating bodies. Other parts of the elastic body show small deformations and thus are assumed to be rigid. Because of the symmetry of the structure, the deformation under *F_x_* is similar to the case of *F_y_*, and the deformation under *M_x_* is similar to the case of *M_y_*. As a result, here we only analyze deformations of the elastic body under *F_x_*, *F_z_*, *M_x_*, *M_z_*.

## 3. Model Development

**Figure 3.**
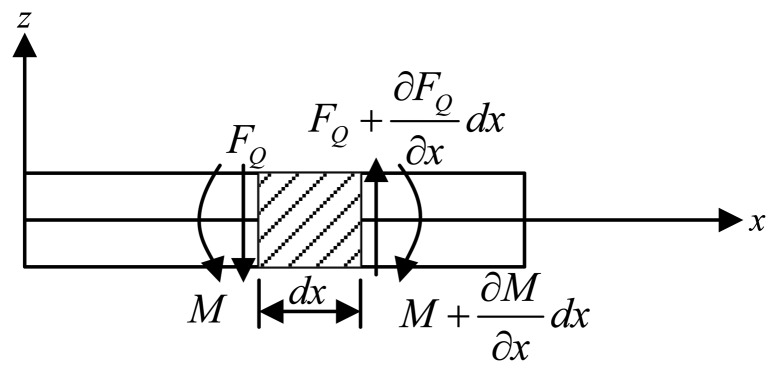
A Timoshenko beam and its infinitesimal section.

Cross-beams elastic bodies are statically indeterminate structures. The responses of cross-beams elastic bodies under forces/torques in different directions involve various complex interactions between cross elastic beams and compliant beams. Deflection characteristics of the above flexural beams under different dimensional forces/torques are analyzed by Timoshenko beam theory [12–14]. The Timoshenko beam theory adopts planar sections hypothesis that all cross sectional areas stay planar after deformation, but the cross sectional areas are not required to stay perpendicular to the deformed axis of the beam. In other words, any cross-section of the beam is treated as an undeformable body that does not allow any displacements other than rigid ones [15]. Thus, the deformation characteristics of the beam are described by two variables, *i.e.*, the translational displacement (*ω*) of any cross-section and the angular displacement (*ψ*) of any cross-section, as is expressed in Equation (1). An infinitesimal section of a Timoshenko beam is illustrated in Figure 3.

(1) $$\{\begin{array}{l} M\;(x)=-EI\frac{d\psi \;(x)}{dx}\\ F_{Q}\;(x)=\text{kGA}\;(\frac{d\omega \;(x)}{dx}-\psi \;(x)) \end{array}$$

where *x* denotes the *x*-coordinate value of any point in the beam as is shown in Figure 3 (it also indicates the distance of the point away from the beginning of the beam), *M* is the bending moment and *F_Q_* is the shear force, *A* represents the cross-sectional area, *E*, *I*, *k*, *G* are the elastic modulus, second area moment, shear coefficient and shear modulus, respectively.

Equation (1), together with force/torque equations of equilibrium for the infinitesimal section depicted in Figure 3, can yield

(2) $$\{\begin{array}{l} \frac{d}{dx}[C\;(\frac{dw\;(x)}{dx}-\psi \;(x))]=0\\ \frac{d}{dx}\;(D\;\frac{d\psi \;(x)}{dx})-C\;(\frac{d\omega \;(x)}{dx}-\psi \;(x))=0 \end{array}$$

where *C* = *kGA,D* = *EI*.

Equation (1) and Equation (2) are two basic formulas of Timoshenko beam theory, from which analytical solutions of *ω*(*x*) and *ψ*(*x*) could be calculated when given enough boundary conditions.

Once the analytical solution of angular displacement *ψ*(*x*) is obtained, the strain value (*ε*) of any point in the beam can be calculated in Equation (3).

(3) $$ɛ\;(x,z)=-z\frac{d\psi \;(x)}{dx}$$

where *z* is *z*-coordinate value of any point in the beam and it also indicates the distance between the point and the neural plane.

In following sub-sections, mechanical models of cross-beams elastic bodies under effects of *F_x_*, *F_z_*, *M_x_*, *M_z_* are established respectively to estimate strain distributions on related cross elastic beams. Overall, the modeling approach in this paper incorporates flexibilities and interactions among cross elastic beams and compliant beams and enables a faithful simulation of deformations. Detailed modeling approach consists of the following steps: (i) establish idealized mechanical model according to deformations of an elastic body under effects of force/torque in each direction; (ii) analyze deformed cross elastic beams and compliant beams utilizing Timoshenko beam theory and obtainable boundary conditions; (iii) determine unknown parameters in derived analytical solutions of *ω*(*x*) and *ψ*(*x*) based on displacement relationships of relative beams and force/torque equations of equilibrium; (iv) derive formulas for strain distributions.

### 3.1. Mechanical Model under F_x_

**Figure 4.**
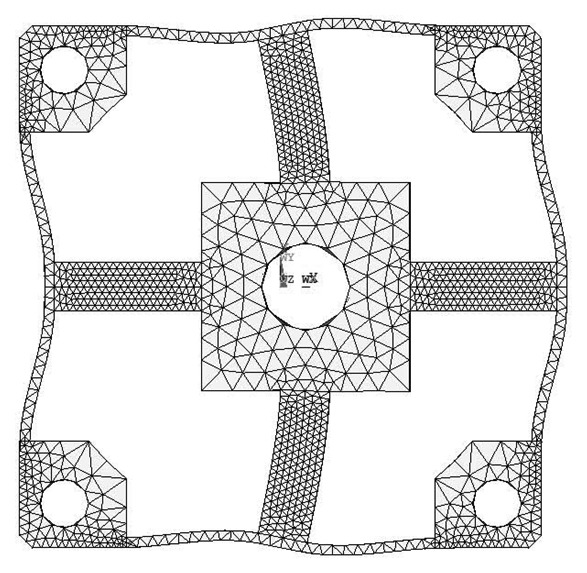
Deformation of an elastic body under *F_x_*.

As is shown in Figure 4, when *F_x_* is loaded, bending occurs on cross elastic beams *EF*, *GH* and compliant beams *AQ*, *AI*, *DJ*, *DK*. Compliant beams *HL* and *HM*, *EN* and *EP* become two elastic supports of beams *EF* and *GH*.

**Figure 5.**
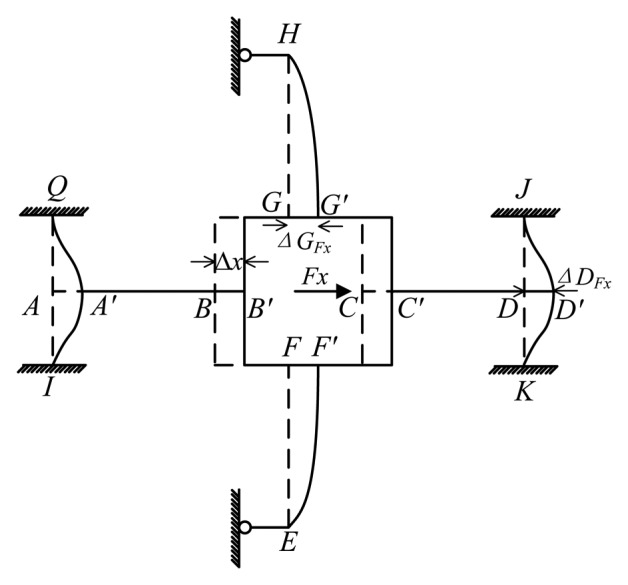
Mechanical model of an elastic body under *F_x_*.

Based on the deformation configuration, the mechanical model of the elastic body is proposed in Figure 5. Compliant beams *HL* and *HM*, *EN* and *EP* are simplified as roller supports *H* and *E*. Other conjunctions are deemed as rigid joints. Thus cross elastic beams *EF*, *GH* are idealized as propped-cantilever beams and compliant beams *AQ*, *AI*, *DJ*, *DK* are idealized as cantilever beams. In addition, it can be easily noticed that axial forces are transmitted through cross elastic beams *AB* and *CD*. Accordingly, beams *AB* and *CD*, which are subjected to axial forces, show negligible tension or compression deformations, and thus could be regarded as rigid bodies. Notations with primes (e.g., *A*′, *B*′, *C*′) indicate displaced positions of original ones (e.g., *A*, *B*, *C*). Δ*x*, Δ*G_Fx_*, and Δ*D_Fx_* represent the displacement of the square convex, the displacement of node *D* in compliant beam *DJ*, and the displacement of node *G* in cross elastic beam *GH*, respectively. Owning to the symmetry of structure, the deflection characteristic of cross elastic beam *EF* is similar to *GH* and the deflection characteristic of compliant beam *DJ* is similar to *DK*, *AQ*, *AI*. Here we only consider cross elastic beam *GH* and compliant beam *DJ*.

Δ*G_Fx_* can be obtained from a combination of Equation (2) and boundary conditions of Equation (2) observed from beam *HG* in Figure 5 as follows

(4) $$\Delta G_{F_{x}}=\frac{l_{2}^{3}F_{GF_{x}}}{3D_{2}}+\frac{l_{2}F_{GF_{x}}}{C_{2}}$$

where *C*_2_ = *kGA*_2_, *D*_2_ = *EI*_2_, *A*_2_ = *b*_2_*h*_2_ is the cross-sectional area of cross elastic beams, $I_{2}=\frac{b_{2}h_{2}^{3}}{12}$, *F_GFx_* is the shear force on cross elastic beam *GH*.

Δ*D_Fx_* is derived from Equation (2) and obtainable boundary conditions from beam *DJ* in Figure 5 as follows

(5) $$\Delta D_{F_{x}}=\frac{l_{1}^{3}F_{DF_{x}}}{12D_{11}}+\frac{l_{1}F_{DF_{x}}}{C_{1}}$$

where *C*_1_ = *kGA*, *D*_11_ = *EI*_1_, *A*_1_ = *b*_1_*h*_1_ is the cross-sectional area of cross elastic beams, $I_{11}=\frac{h_{1}b_{1}^{3}}{12}$, *F_DFx_* is the shear force on compliant beam *DJ*.

According to the geometric characteristic of the deformation as is depicted in Figure 5, the displacement equation of the elastic body can be written as below

(6) $$\Delta x=\Delta G_{F_{x}}=\Delta D_{F_{x}}$$

The force equations of equilibrium of the square convex is derived as below

(7) $$4F_{DF_{x}}+2F_{GF_{x}}=F_{x}$$

The combination of Equation (2) through Equation (7) leads to following

(8) $${ɛ}_{F_{x}}\;(x,z)=-z\;(\frac{F_{GF_{x}}}{D_{2}}x-\frac{l_{2}F_{GF_{x}}}{D_{2}})$$

where $F_{GF_{x}}=\frac{\frac{l_{1}^{3}}{12D_{11}}+\frac{l_{1}}{C_{1}}}{2\;(\frac{l_{1}^{3}}{12D_{11}}+\frac{l_{1}}{C_{1}})+4\;(\frac{l_{2}^{3}}{3D_{2}}+\frac{l_{2}}{C_{2}})}F_{x}$, *ε_Fx_* represents the strain value of any point on *GH*.

### 3.2. Mechanical Model under F_z_

**Figure 6.**
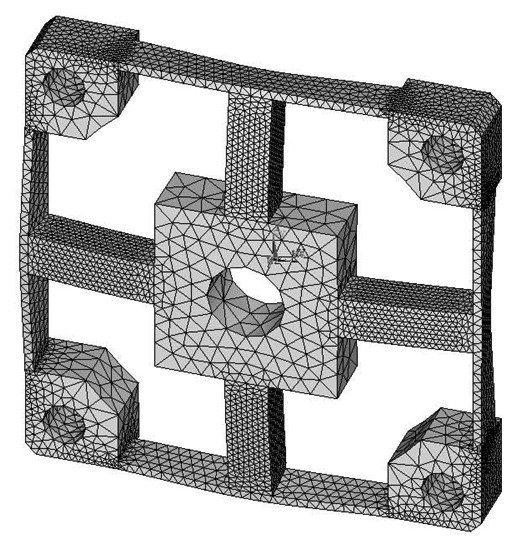
Deformation of an elastic body under *F_z_*.

**Figure 7.**
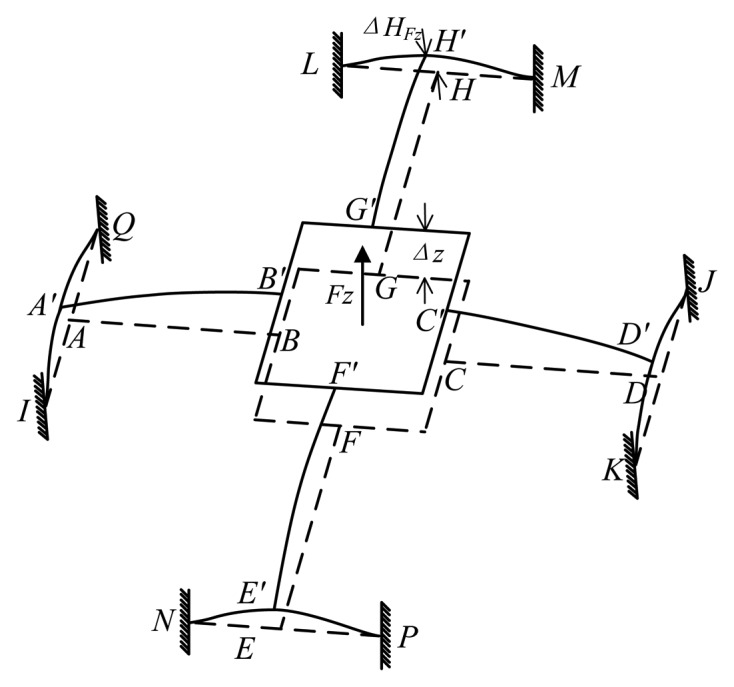
Mechanical model of an elastic body under *F_z_*.

The deformation of the elastic body in the case of *F_z_* is shown in Figure 6. Bending deformations occur on all cross elastic beams and compliant beams. Compliant beams *AI* and *AQ*, *DK* and *DJ*, *EN* and *EP*, *HL* and *HM* turn into four elastic supports for cross elastic beams *AB*, *CD*, *EF*, *GH*, respectively. The idealized mechanical model is established in Figure 7 according to geometric characteristics of the deformation. All cross elastic beams are simplified as propped-cantilever beams and all compliant beams are simplified as fixed beams. Δ*z* respects the displacement of the square convex, Δ*HF_z_* respects the displacement of node *H* in compliant beam *HM*. Owning to the symmetry of the structure, here we only derive equations for analyzing cross elastic beam *GH* and compliant beam *HM*.

Let Δ*G_Fz_* represent the vertical distance between node *G*′ and node *H*′ in Figure 7. Δ*G_Fz_* can be derived from Equation (2) and boundary conditions of Equation (2) obtained from beam *GH* in Figure 7 as below

(9) $$\Delta G_{F_{z}}=-(\frac{l_{2}^{3}}{3D_{2}}+\frac{l_{2}}{C_{2}})\;F_{GF_{z}}$$

where *F_GFz_* represents the shear force in beam *GH*.

Similarly, Δ*H_Fz_* can be calculated from Equation (2) and obtained boundary condition from beam *HM* in Figure 7 as

(10) $$\Delta H_{F_{z}}=-(\frac{l_{1}^{3}}{12D_{12}}+\frac{l_{1}}{C_{1}})\;F_{HF_{z}}$$

where $D_{12}=EI_{12},I_{12}=\frac{b_{1}h_{1}^{3}}{12}$, *F_HFz_* represents the shear force of compliant beam *HM*.

Additionally, the force equations of equilibrium of the elastic body lead to

(11) $$F_{z}=-4F_{GF_{z}}=-8F_{HF_{z}}$$

Besides, according to geometric characteristics of the deformation under *F_z_*, the displacement (Δ*z*) of the square convex under *F_z_* can be obtained as

(12) $$\Delta_{z}=\Delta G_{F_{z}}+\Delta H_{F_{z}}$$

Let *ε_Fz_* represent strain of any point in cross elastic beam *GH*, analytical expressions of *ε_Fz_* can be calculated from Equation (2) and Equation (3), Equation (9) through Equation (12) as follows

(13) $${ɛ}_{F_{z}}\;(x,z)=-z\;(\frac{F_{z}}{4D_{2}}x-\frac{l_{2}F_{z}}{4D_{2}})$$

### 3.3. Mechanical Model under M_x_

**Figure 8.**
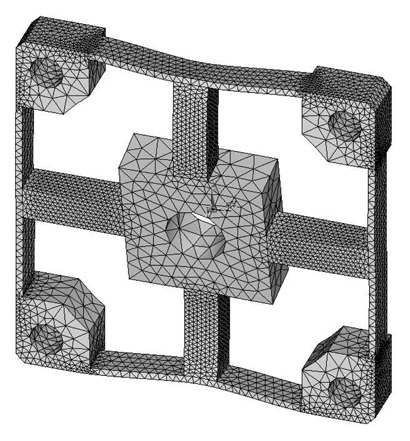
Deformation of an elastic body under *M_x_*.

The deformation of the cross-beams elastic body under *M_x_* is shown in Figure 8. Bending deformations occur on cross elastic beams *EF* and *GH*. Torsional deformations occur on cross elastic beams *AB* and *CD*.

**Figure 9.**
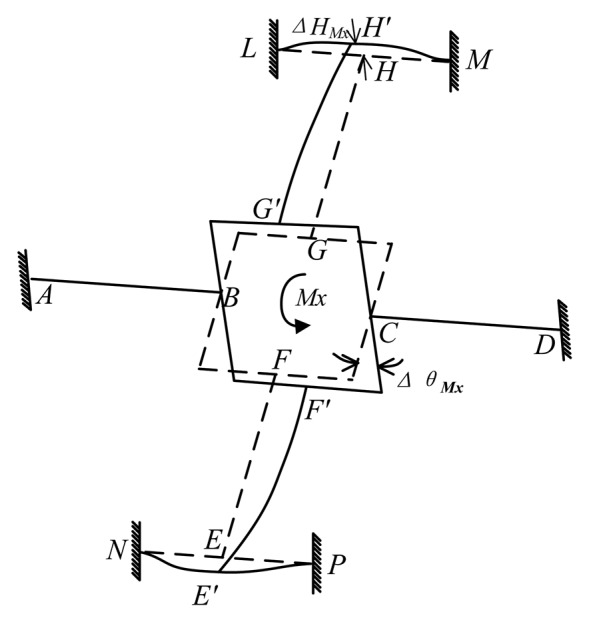
Mechanical model of an elastic body under *M_x_*.

As is shown in Figure 8, compared with the rotation of the square convex with respect to *x*-axis in the global coordinate, there are very small rotations and translations in compliant beams *AQ* and *AI*, *DJ* and *DK*. As for beams *AB* and *CD*, there are strong reaction moments from beams *AQ*, *AI*, *DJ*, *DK*. As a result, compliant beams *AQ* and *AI*, *DJ* and *DK* are idealized as two fixed supports for beams *AB* and *CD*, respectively. Compliant beams *HL* and *HM*, *EN* and *EP* are idealized as beams with fixed supports. Consequently, for cross elastic beams *EF* and *GH*, nodes *H* and *E* can be simplified as roller supports. The mechanical model of the elastic body under *M_x_* is illustrated in Figure 9. The notation Δ*θ_Mx_* describes the angle of rotation of the square convex with respect to *x*-axis of the global coordinate and the notation Δ*H_Mx_* describes the displacement of *H*. The bending of beam *EF* is the same as the case of beam *GH*, the bending of beam *HM* is the same as the case of beams *HL*, *EP*, *EN*. The torsion of beam *AB* is the same as the torsion of beam *CD*. Accordingly, here we only consider deformation conditions of beams *AB*, *GH* and *HM*.

The torsion angle (*α*) of cross elastic beam *AB* can be obtained according to the equation of torsion angle for rectangular beams [16–18] in Equation (18)

(14) $$\alpha =\frac{M_{BM_{x}}l_{2}}{GI_{t}}$$

where *M_BMx_* represents the applied torque on beam *AB*, $I_{t}=\beta b_{2}^{3}h_{2}$, *β* is a coefficient for rectangular beams that is relevant to $\frac{h_{2}}{b_{2}}$. When $\frac{h_{2}}{b_{2}}=1$, *β* = 0.141.

Δ*r_Mx_* respects the angular displacement of the overlapping surface between the square convex and the cross elastic beam *GH* (hereinafter referred to as “beginning surface” of cross elastic beam *GH*) with respect to *x*-axis in global coordinate. It can be calculated by Equation (2) and boundary conditions of Equation (2) obtained from deformed characteristics of beams *GH* and *HM* in Figure 8 as follows

(15) $$\Delta r_{M_{x}}=\frac{3C_{2}D_{2}\Delta H_{M_{x}}-C_{2}F_{GM_{x}}l_{2}^{3}-3D_{2}F_{GM_{x}}l_{2}}{3C_{2}D_{2}\;(l_{2}+r)}$$

where $\Delta H_{M_{z}}=-F_{GM_{x}}\;(\frac{l_{1}^{3}}{12D_{12}}+\frac{l_{1}}{C_{1}})$, *F_GMx_* represents the shear force on beam *GH*.

Geometric characteristics of the deformed elastic body under *M_x_* can yield

(16) $$\Delta \theta_{M_{x}}=\Delta r_{M_{x}}=\alpha$$

Force/torque equations of equilibrium of the elastic body lead to

(17) $$2M_{BM_{x}}+2M_{GM_{x}}-2F_{GM_{x}}r=M_{x}$$

where *M_GMx_* is the bending moment on the beginning surface of beam *GH*, $F_{GM_{x}}=\frac{M_{GM_{x}}}{l_{2}}$.

Combinations of Equation (2) through Equation (3), Equation (14) through (17) lead to

(18) $${ɛ}_{M_{x}}\;(x,z)=-z\;(-\frac{F_{GM_{x}}}{D_{2}}x+\frac{l_{2}F_{GM_{x}}}{D_{2}})$$

where *ε_Mx_* represents the strain value of any point on beam *GH*, *F_GMx_* can be calculated in the following equation:

(19) $$F_{GM_{x}}=\frac{-3C_{2}D_{2}l_{2}\;(l_{2}+r)\;M_{x}}{W}$$

where $W=3C_{2}D_{2}\;[2l_{2}{\;(l_{2}+r)}^{2}+GI_{t}\;(\frac{l_{1}^{3}}{12D_{12}}+\frac{l_{1}}{C_{1}})]+2GI_{t}\;(C_{2}l_{2}^{3}+3D_{2}l_{2})$.

### 3.4. Mechanical Model under M_z_

**Figure 10.**
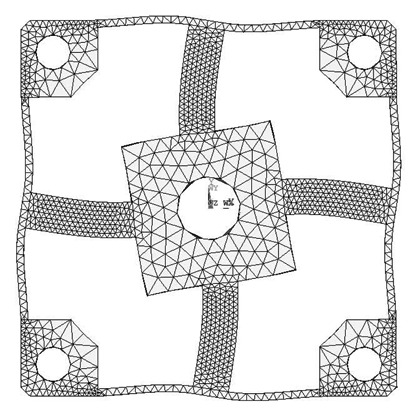
Deformation of an elastic body under *M_z_*.

**Figure 11.**
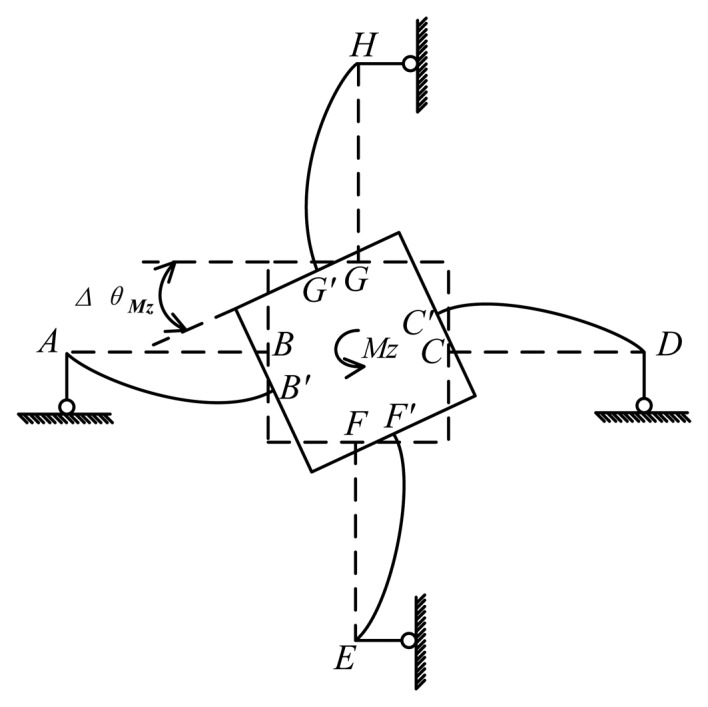
Mechanical model of an elastic body under *M_z_*.

Under the effect of *M_z_*, bending deformations happen on all cross elastic beams of the elastic body as is shown in Figure 10. Compliant beams *AQ* and *AI*, *DJ* and *DK*, *EN* and *EP*, *HL* and *HM* become four elastic supports of beams *AB*, *CD*, *EF*, *GH*, respectively Here we take cross elastic beam *CD* for example. The right end of beam *CD* is free to rotate and translate along x-axis in global coordinate, compared with small translations along y-axis in global coordinate. Taking into consideration the strong reaction force perpendicular to beam *CD*, the reaction moment and force of *D* along cross beam *CD* could be ignored. Hence, the four elastic supports can be simplified as four roller supports, *i.e.*, nodes *A*, *D*, *E*, *H* in the proposed mechanical model of the elastic body under *M_z_*, as is depicted in Figure 11. As a result, four cross elastic beams are idealized as propped-cantilever beams. The notation Δ*θ_Mz_* is the angle of rotation of the square convex with respect to z-axis in the global coordinate under *M_z_*.

The force/torque equation of equilibrium of the square convex under *M_z_* can be written as

(20) $$-4F_{GM_{z}}r-4M_{GM_{z}}=M_{z}$$

where *F_GMz_* and *M_GMz_* are the shear force and bending moment of the beginning surface of *GH* respectively, *M_GMz_* = *F_GMz_l*_2_. Equation (2) and Equation (20), together with boundary conditions of Equation (2) obtained from beam *GH* in Figure 11, can yield, after integration

(21) $$\Delta \theta_{M_{z}}=\frac{l_{2}^{3}C_{2}+3D_{2}l_{2}}{12C_{2}D_{2}{\;(r+l_{2})}^{2}}\;Mz$$

Similarly, equations for analyzing strain distribution of *GH* can be derived as

(22) $${ɛ}_{M_{z}}\;(x,z)=-z\;(-\frac{F_{GM_{z}}}{D_{2}}x+\frac{l_{2}F_{GM_{z}}}{D_{2}})$$

where $F_{GM_{z}}=-\frac{M_{z}}{4r+4l_{2}}$.

## 4. Validation and Results

### 4.1. FEA Simulations

The analytical solutions proposed in the previous section are validated against the FEA simulation results by four different cross-beams elastic bodies with various geometric dimensions. The material of the elastic bodies is defined as 2A12 Duralumin, in which the elastic modulus (E) is 72 GPa, the Poisson's ratio is 0.33 and the Yield strength is 380 MPa. Span-to-depth ratios of the cross elastic beams of the four elastic bodies range from 3:1 to 6:1. According to the principle of Timoshenko beam theory, in general, the higher the span-to-depth ratios are, the more accurate the proposed analytic solutions will be, because the planar sections hypothesis as mentioned in the previous section will be less accurate when the span-to-depth ratios of the Timoshenko beam decrease [19]. All other geometric parameters and measurement ranges are varied within expected practical ranges. Table 1 presents a summary of the four elastic bodies including geometric details as well as measurement ranges.

FEA simulations are calculated in ANSYS 11.0, while the 3-D, 20 nodes “Solid95” is chosen as the element type of FEA models for its characteristic of high tolerance with respect to irregular shapes without as much loss of accuracy [20]. Taking the Example 1 for instance, the mesh-size of cross elastic beams is defined as twenty equal parts in the height/width-direction and thirty equal parts in the length-direction. The mesh-size of compliant beams is defined as fifteen equal parts in the height/length-direction and bisection in the width-direction. Mesh lever of other examples are similar to the case of Example 1. For degree-of-freedom (DOF) constrains, displacements and rotations of surfaces of four location holes in all directions are restricted to zero. External force/torque in positive full-scale value of each dimension is loaded on related locations in square convex.

Deformation conditions under each single force/torque are analyzed. Strains of nodes on the lateral surface or upper surface, which corresponds to the surface for strain gauge locations of each force/torque component, on cross elastic beam *GH* are recorded.

### 4.2. Calibration Experiment

**Figure 12.**
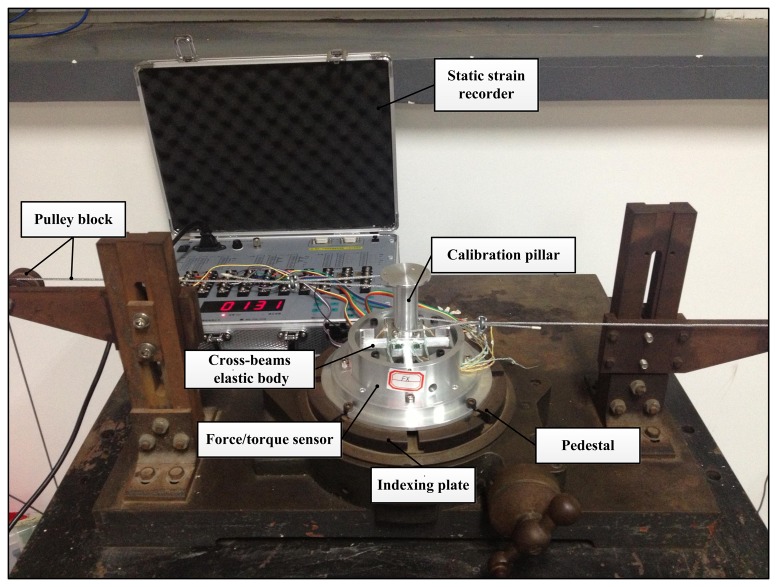
The calibration experiment setup.

Besides FEA simulations, calibration experiments are implemented to enhance validation of the correctness of proposed analytic solutions. Special calibration experiments are designed to test strains of strain-gauge-locations in cross elastic beams under external forces and torques. The experiment platform of the calibration experiments is shown in Figure 12. A cross-beams six-axis force/torque sensor is mounted on a rotatable indexing plate to guarantee directions of loading forces and torques.

The force/torque sensor is fabricated from 2A12 Duralumin. The calibration pillar is fixed on the cross-beams elastic body to facilitate force/torque loading process in calibration experiments. Other parts like pedestal are used for supporting and sealing. Under white protective coatings, twenty-four uniaxial foil strain gauges are attached to cross elastic beams with special adhesives for strain gauges bonding. Six Wheatstone bridges are constructed for the measurement of force/torque in six dimensions. Measurement ranges and geometric dimensions of the sensor are the same as Example 1 in Table 1. Strain gauges for force measurements are pasted 6 mm away from beginning surfaces, strain gauges for torque measurements are pasted 12 mm away from beginning surfaces.

The six Wheatstone bridges on cross elastic beams are connected to a static strain recorder. The static strain recorder provides more than six input channels and a direct reading LED display. It is capable of displaying strains of strain gauges when connected to quarter-, half-, and full- bridge strain-gauge circuits. The resolution of the strain recorder is 1 *μϵ*, the measurement range is ±19,999 *μϵ*, and the accuracy is 0.5% ± 3*μϵ*. These features meet requirements of the calibration experiments in our work.

During calibration process, force or torque in each dimension is calibrated separately by a series of standard loads. Weights and pulley blocks are utilized for force/torque loading [21]. Loading forces in *X* direction are generated by a unilateral pulley block and weights while indexing plate is rotated to adjust the loading direction. Loading forces in *Z* direction are generated by weights, which are directly put on the calibration pillar. Loading torques in *X* direction are created by a pair of horizontal forces. One force is applied on the upper part of the calibration pillar, and the other force is applied on the lower part in opposite direction. Loading torques in *Z* direction are generated by applying one horizontal force on the right side of the calibration pillar and the other equal and opposite force on the left side. The calibration range of *Fx* is from −100 N to +100 N with an incremental step of 10 N, the calibration range of *Fz* is from −100 N to 0 N with an incremental step of 10 N. The calibration range of *Mx* is from −10 Nm to +10 Nm with an incremental step of 1 Nm, the calibration range of *Mz* is from 0 Nm to 12 Nm with an incremental step of 0.8 Nm.

Besides calibration of one-dimensional force/torque, three groups of combined loads are applied during the calibration experiments. In the first group, various loading forces in *Z* direction are applied while a constant force is applied in *X* direction. In the second group, various loading torques in *X* direction are applied while a constant force is applied in *Z* direction. In the third group, various one-dimensional loading forces are applied along the bisector between *X* and *Y* directions.

### 4.3. Validation of the Analytical Model

As for the proposed analytic solutions, derived Equations (8), (13), (18) and (22) are utilized to compute strains of elastic bodies under *F_x_*, *F_z_*, *M_x_*, *M_z_* respectively. All calculations are done in MATLAB 2010b while the shear coefficient (*k*) is defined to be 1.0 and the shear modulus (*G*) can be calculated as 27 GPa. Other material properties like the elastic modulus (*E*) are defined the same as the case in FEA simulations.

**Figure 13.**
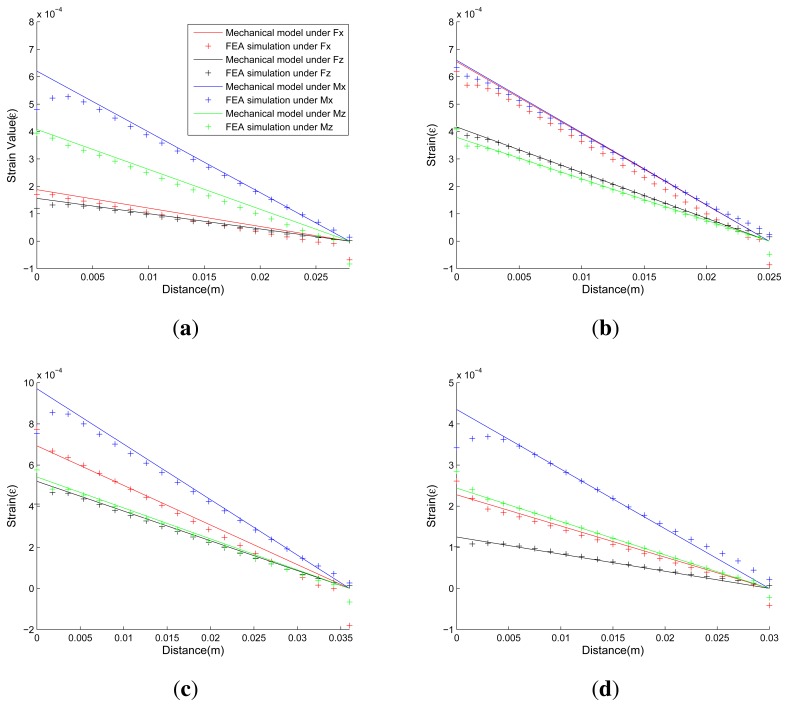
Comparison of analytical solutions and FEA simulations. (a) Example 1; (b) Example 2; (c) Example 3; (d) Example 4.

Figure 13 presents comparisons of strains calculated by the proposed analytical solutions and the FEA simulation results under applied force/torque in each dimension. In each sub-figure of Figure 13, two kinds of data are presented. The first kind of data, which are depicted by colored lines, represent strains calculated from Equations (8), (13), (18) and (22) in the proposed mechanical models. The second kind of data, which are depicted by colored points, represent strain calculated from FEA simulations. The colors red, black, blue, and green indicate that the elastic body is under effect of *F_x_*, *F_z_*, *M_x_*, and *M_z_* respectively. The horizontal axis represents the distance of any node in selected surface away from the beginning surface, corresponding to parameter x in the mechanical models. The vertical axis represents the strain value of the node, corresponding to parameters *ε_Fx_,ε_Fz_,ε_Mx_* and *ε_Mz_* in the mechanical models.

Good agreement is shown between proposed analytical solutions and FEA solutions. The FEA simulated strains of nodes, which locate near the beginning or the end surface of the cross elastic beam, may drift noticeably from the strain value predicted by the proposed mechanical models. The discrepancy in the responses can be attributed to the stress concentration due to sharp corners in the mechanical structure, which is not taken into account in the proposed mechanical models. However, in practice, the stress concentration can be largely eased by adding fillets in the sharp corners and efficiently avoided by keeping some space between strain gauges and the sharp corners. As a result, the deviations caused by stress concentration are insignificant and can be ignored.

**Figure 14.**
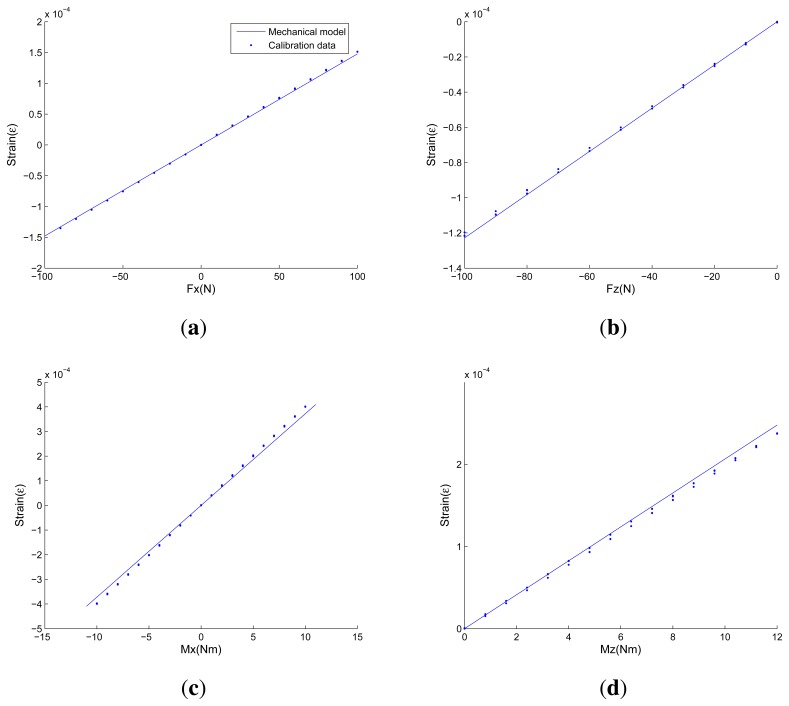
Comparison of analytical solutions and calibration data under one-dimensional force/torque. (a) Strains on strain-gauge-locations under *F_x_*; (b) Strains on strain-gauge-locations under *F_z_*; (c) Strains on strain-gauge-locations under *M_x_*; (d) Strains on strain-gauge-locations under *M_z_*.

Figure 14 and Table 2 show comparisons between strains of strain-gauge-locations obtained from the calibration experiments and strains calculated from proposed analytical solutions under one-dimensional force/torque and combined loads, respectively. In each sub-figure of Figure 14, blue points represent calibration data obtained from calibration experiments, and the blue line represents line drawn of strains calculated from proposed analytical solutions. The horizontal axis represents a series of standard loading forces/torques, while the vertical axis represents strains of strain-gauge-locations. In Table 2, strains obtained from calibration data and proposed analytical solutions under three groups of combined loads are presented.

As is demonstrated in Figure 14 and Table 2, strains estimated from proposed analytical solutions are in close agreement with calibration data. The mechanical models are able to estimate strain distributions, which are the key response parameters and features of cross-beams elastic bodies in high precision. When one-dimensional force/torque is applied, the prediction error of the proposed mechanical models is under 10%. The observed deviations occur for various reasons, like machining accuracy, modeling idealizations of the Timoshenko beam and elastic bodies. When combined loads are applied, the prediction error is under 20%. Compared with the accuracy under one-dimensional force/torque, the increased errors come from coupling errors, which can be reduced by decoupling algorithms [21–23]. Note that in order to ensure proper operation and validate the correctness of measurements, even those sensors that are fabricated by most sophisticated instruments need to be calibrated from time to time. According to empirical data, the expected calculated strains at the attachment locations of foil strain gauges under effects of full scale values should be 1.0 × 10^−4^ ∼ 9.9 × 10^−4^ to ensure both sensitivity and strength. As a result, the accuracy of the proposed mechanical models is high enough in general.

Having gained confidence in the reliability of the detailed mechanical models, herein we examine another crucial criterion, i.e., the processing speed for calculations. To evaluate the processing speed, the calculation time of the proposed mechanical models and the FEA with respect to the above four examples are recorded respectively. The whole process is repeated 5 times. All calculations are made on a Windows XP Inter(R) Core (TM) 2 Duo CPU, 2.8 GHz processor with 2.0 GB RAM. The FEA simulations are carried out by means of a prewritten program in APDL (ANSYS Parametric Design Language).

Table 3 shows a summary of the averaged calculation time. For each example, the elapsed time of preprocessing and solution steps in FEA under *F_x_*, *F_z_*, *M_x_*, *M_z_* are recorded separately, and the total calculation time is a summarization of the four elapsed time. It can be easily found that the total calculation time is reduced from more than 1,000 seconds to less than 0.0005 second by the proposed analytical solutions. The calculation time of the proposed analytical solutions is much less than that for FEA simulations. The proposed analytical solutions are proven to be a fast prediction tool for strains of cross-beams elastic bodies for six-axis force/torque sensors.

## 5. Conclusions

This paper proposes analytical solutions for fast estimating strain distributions of cross-beams six-axis force/torque sensors by means of mechanical modeling. The mechanical models are based on Timoshenko beam theory, which accounts for shear deformation and rotational inertial effects. Formulas are derived via second order differential equations in terms of translational and angular displacement while their boundary conditions are obtained from deformations of elastic bodies under external force/torque in each dimension. The strain distributions on cross elastic beams of elastic bodies are formulated. Closed-form expressions of strains in terms of complicated geometric parameters, loads and material properties are obtained.

The models are validated by FEA simulations and calibration experiments, and the proposed models are found to provide reliable analytical solutions for evaluating strains. The application scope of the proposed mechanical models is wide. The precondition is that the span-to-depth ratio of cross elastic beams should be higher than 3:1. Compared with FEA simulations, the calculation time is greatly reduced without compromising accuracy in our work, which will be advantageous in computer simulation of whole design process of cross-beams six-axis force sensors. The proposed analytic solutions of cross-beams elastic bodies can also be used to verify the correctness of FEA simulations.

## Figures and Tables

**Table 1.** Summary of four elastic bodies for testing.

**Reference**	**Compliant Beam (mm)**			**Cross Elastic Beam (mm)**			**Square Convex (mm)**	**Measurement Range (N/Nm)**			
			
	***l****_1_*	***h****_1_*	***b****_1_*	***l****_2_*	***h****_2_*	***b****_2_*	***r***	***F****_x_*	***F****_z_*	***M****_x_*	***M****_z_*
Example 1	17.9	7.2	2	28	7.2	7.2	18	100	100	10	12
Example 2	15.5	5	1	25	5	5	8	100	100	3	3
Example 3	25	8	1.5	36	6	6	15.25	150	150	8	8
Example 4	26.5	10	2	30	10	10	21.25	200	200	20	20

**Table 2.** Comparison of analytical solutions and calibration data under combined loads.

**Strain Values(*μϵ*)**	**Fx = 40 N**		**Fz = −40 N**		**Fx = Fy**	
		
	**Fz = −20 N**	**Fz = −80 N**	**Mx = 2 Nm**	**Mx = −6 Nm**	**Fy = 28.3 N**	**Fy = 56.57 N**
Calibration Data	*ε_F_x__* = 56.9	*ε_F_x__* = 57.0	*ε_F_x__* = −56.8	*ε_F_z__* = −61.5	*ε_F_x__* = 41.5	*ε_F_x__* = 82.6
	*ε_F_z__* = −31.1	*ε_F_z__* =−122.7	*ε_M_x__* = 78.2	*ε_M_x__* =−233.5	*ε_F_y__* = 40.9	*ε_F_y__* = 82.2

Mechanical Model	*ε_F_x__* = 59.1	*ε_F_x__* = 59.1	*ε_F_z__* = 49.1	*ε_F_z__* = 49.1	*ε_F_x__* = 41.8	*ε_F_y__* = 83.6
	*ε_F_z__* = −24.6	*ε_F_z__* = −98.2	*ε_M_x__* = 74.4	*ε_M_x__* = −223.2	*ε_F_x__* = 41.8	*ε_F_y__* = 83.6

**Table 3.** Averaged calculation time (in second) of mechanical models and FEA.

**Elapsed Time**	**Mechanical Model (total calculation time)**	**FEA**				

		**Fx**	**Fz**	**Mx**	**Mz**	**Total Calculation Time**
Example 1	0.000393	288.9	293.9	303.3	304.7	1,190.8
Example 2	0.000464	335.4	346.8	342.7	349.1	1,374.0
Example 3	0.000469	246.1	248.0	253.5	252.9	1,000.5
Example 4	0.000488	468.4	470.2	472.9	468.6	1,880.1
